# Number of Students in Some of the Eastern Medical Colleges

**Published:** 1855-12

**Authors:** 


*Number of Students in some of the Eastern Medical Colleges.*—According to the New York *Medical Gazette*, the whole number of Medical students atttending the colleges in that city is about four hundred and thirty-five. They are distributed as follows: the University School 200; the College of Physicians and Surgeons 160, and the New York Medical College 75. The December number of the New Jersey *Reporter*, says the aggregate number of students attending the schools in Philadelphia this winter is less than heretofore. The number in the Pennsylvania University has fallen off, about 50; in the Jefferson School about 100; while the number attending the Pennsylvania and Philadelphia Colleges, has moderately increased.

